# Digital Media Exposure and Predictors for Screen Time in 12-Month-Old Children: A Cross-Sectional Analysis of Data From a German Birth Cohort

**DOI:** 10.3389/fpsyt.2021.737178

**Published:** 2021-11-29

**Authors:** Kira Durham, David Wethmar, Susanne Brandstetter, Birgit Seelbach-Göbel, Christian Apfelbacher, Michael Melter, Michael Kabesch, Sebastian Kerzel, The KUNO Kids Study Group, Andreas Ambrosch

**Affiliations:** ^1^Department of Pediatric Pneumology and Allergy, University Children's Hospital Regensburg (KUNO), Hospital St. Hedwig of the Order of St. John, University of Regensburg, Regensburg, Germany; ^2^University Children's Hospital Regensburg (KUNO), Hospital St. Hedwig of the Order of St. John, University of Regensburg, Regensburg, Germany; ^3^WECARE Research and Development Campus Regensburg, Hospital St. Hedwig of the Order of St. John, University of Regensburg, Regensburg, Germany; ^4^University Department of Obstetrics and Gynecology, Hospital St. Hedwig of the Order of St. John, University of Regensburg, Regensburg, Germany; ^5^Institute of Social Medicine and Health Systems Research, Otto von Guericke University Magdeburg, Magdeburg, Germany; ^6^Department of Pediatrics, University Children's Hospital Regensburg (KUNO), Hospital St. Hedwig of the Order of St. John, University of Regensburg, Regensburg, Germany

**Keywords:** digital media, screen time, infants, young children, media exposure

## Abstract

**Background:** Early exposure to digital media may affect the physical and cognitive development in young children. The *American Academy of Pediatrics* and national guidelines recommend no digital media use at all under the age of 18 months. The aim of our study was to determine the actual exposure to digital media in 12-month-old infants and to reveal potential risk factors for screen time.

**Methods:** In this prospective cross-sectional survey, data was collected from the KUNO Kids birth cohort study using parent-report questionnaires regarding the media exposure of the study child. We determined age at first contact with different digital media, mean screen time on an average weekday, and the influence of major demographic and socioeconomic factors.

**Results:** Data for screen time analysis was available for 630 children. In summary, 45% of children had already been exposed to digital media by their first birthday. The most frequent first digital media exposure was the TV (33.0%) followed by smartphones (16.9%), both most commonly exposed to around the age of 8 months. On a regular weekday, 20% of the children spent 0.5–1 h in front of a TV and 9% were exposed to a smartphone for the same time frame, compared to 31% of joint parent-child media use. Predictors for screen time were having one sibling, less living space per person, and excessive TV use in the household, the latter of which doubled the chance of the child being exposed to digital media.

**Conclusion:** A proportion of 10% of 1-year-old children were already regularly exposed to digital media. The TV remains the most predominant device but new media, particularly smartphones, might be catching up. Our study provides further support that family TV time is a major predictor of infant screen time. Pediatric recommendations should be re-evaluated in the light of the actual exposure to digital media already in infancy and parents should be proactively counseled regarding possible effects on child development.

## Introduction

Compared to twenty years ago, the digital media environment has flourished (1, 2). Children are growing up with both traditional electronic media (television) and new interactive media, such as entertainment gadgets (game consoles, video/DVD/Blu-ray players, and tablets), work devices (personal computers and laptops), multi-functional devices (smartphones) and not to forget electronic book readers. Infants today may learn to use digital media before they learn to walk.

However, premature contact and overuse of digital media may disturb an infant's development in the first year of life. The *American Academy of Pediatrics* (3) and national guidelines (4) recommend no electronic media use in children under 18 months, other than video-chatting with family members, as children younger than 2 years of age do not benefit from 2D screen media. For children 2–5 years old, a daily amount of less than one hour is advised (3). Yet, in Europe, approximately one third of 2–10-year-old children fail to meet current recommendations (5).

Media exposure can affect child development directly (e.g., consumption of violent media content) or indirectly (e.g., displacement of time) (6). Sleep is paramount for child development (7, 8), and evidence shows that increased daily screen time is associated with shorter sleep per night (9, 10). The displacement of sleeping (9) and physical activity (11), and especially for infants, playing and interacting with their parents or other family members (12) can have multiple negative consequences (11, 13–15).

While parental media use has been proven to be a pivotal predictor for child media use (2, 16), other risk factors must be considered, including child sex, age, hyperactive and sedentary behavior, as well as parental age, BMI, and education. Availability of digital technology in the household, family size and income have also been associated with the amount of electronic media consumption (17).

In the light of this data, it is crucial to collect information on the actual digital media exposure of today's young children. Therefore, analyzing data from a birth cohort, we examined the amount of digital media use in 12-month-old German infants and we determined factors predicting individual screen time.

## Materials and Methods

### General Study Design of KUNO Kids Birth Cohort

The KUNO Kids Health Study aims to evaluate a wide array of factors and determinants of child health and development in a holistic approach (18). All pregnant women presenting for their pre-birth check-up and women post-delivery at the Obstetrics Department of the University Regensburg (Bavaria), Germany, were invited to participate. The actual inclusion into the study took place during the first 48 h after delivery. General exclusion criteria for the birth cohort were inadequate German language skills, outpatient birth and maternal age less than 18 years. In families with twins, only one child per family was included. Participation was voluntary and all participants provided written informed consent. The study was approved by the Ethics Committee of the University Regensburg (file number: 14-101-0347) (18).

### Data Collection for Digital Media Exposure Study

Regarding the study question, data was obtained at the child's age of 12 months, in the period of June 2015 to January 2019. For this purpose, the families received questionnaires via mail shortly before the first birthday of the child. Parents completed a questionnaire each, as well as one for the participating child and, if present, for siblings. To reduce recall bias, we excluded 299 questionnaires that were returned later than two months after the child's first birthday.

### Outcome Variables

Using five standardized questions with a total of 23 items, parents were asked to estimate the amount of time digital media devices were used by their child on an average weekday. Digital media included TV, Blu-ray/DVD/Video, PC, tablet, smartphone, game console, and, as a reference, books. In addition, parents were asked at what age their child first used various digital media devices, whether their child had a TV in its bedroom, and how long the family TV was running on a typical weekday.

### Exposure Variables

Demographic information was gathered from questionnaires directed specifically to the mother or father, sent a few days after delivery, after four weeks and at the child's first birthday. Following risk factors for child media exposure were considered: sex of the child, number of siblings, mother's age at delivery, overweight of one or both parents, single-parent household, high family TV time (delineated as equal or more than 2–3 h per day) and living space per person less than the calculated median (determined by dividing the household size (m^2^) by the number of household members). Further sociodemographic factors were assessed, including parents' professional qualification on a four parameter scale (“None”: no finished education, not in professional education, “Low”: student or still in professional education, “Medium”: completed education, under university level, “High”: university education), whether parents had been employed during the first year of the child's life, and parents' nationality (both German nationals, one parent foreign national, both foreign nationals). We assessed the subjective social status (SSS) using the *MacArthur Scale*, which is recommended for use in industrialized countries such as Germany (19).

### Statistical Analysis

The reported child's regular daily use of digital media devices was defined as screen time. We then dichotomised the variable *screen time* to group infants with absolutely no screen time compared to those with screen time, as per the AAP guidelines for one-year-old children (3). After excluding questionnaires with a response time lag later than 2 months, a descriptive analysis of *screen time* and sociodemographic data was conducted. Using *screen time* as the dependent variable and the above-mentioned risk factors as independent variables, we ran a univariable binary logistic regression with odds ratio and 95% confidence intervals. To analyse the importance of the predictors relative to each other, we ran a multivariable binary logistic regression, including only those predictors with *p* < 0.2 in the univariable logistic regression. All statistical analyses were performed with IBM SPSS Statistics ® Version 23. Figures were plotted with GraphPad Prism ® 6.07 (La Jolla, USA).

## Results

### Sample Characteristics

Data from the KUNO Kids questionnaires for screen time analysis was available for 630 children. Sociodemographic information was available for 577 families. The detailed characteristics of the sample population are outlined in Table 1. With the birth cohort based in a hospital in South-east Bavaria, most parents were German nationals (86.4%) and had a medium to high professional qualification (98.0% of mothers and 97.7% of fathers). 49.7% of our sample were girls and 60.8% of the children were first-born. Mother's age at delivery was between 22 and 45 years (mean ± SD: 34.7 ± 4.0 years). 38.0% of the mothers were overweight at the time of the survey. The subjective social status on the MacArthur Scale, as noted by both mothers and fathers, was 7 points (mean: 6.9 points and 7.0 points, respectively). Most of the fathers had been employed during the first year of their child's life (97.6%).

**Table 1:** Characteristics of sample population.

		**N**	**Participants**	**Percentage**
Infants	Sex female	577	287	49.7
	First-born	574	349	60.8
	One sibling	574	183	31.9
	Two or more siblings	574	42	7.3
Mothers	Age at delivery^a^ in years (mean) (SD)	571	34.7 (4.0)	
	Overweight^b^	495	188	38.0
	Subjective social status^c^ (mean) (SD)	559	7.0 (1.3)	
	Single parent	567	6	1.1
	*Professional qualification* ^d^			
	None	562	4	0.7
	Low	562	7	1.2
	Medium	562	279	49.6
	High	562	272	48.4
	Employed during the child's first year of life	571	156	27.3
Fathers	Overweight^b^	480	326	32.1
	Subjective social status ^c^ (mean) (SD)	560	6.9 (1.4)	
	*Professional qualification* ^d^			
	None	521	4	0.8
	Low	521	8	1.5
	Medium	521	252	48.4
	High	521	257	49.3
	Employed during the child's first year of life	553	540	97.6
Home environment	One or both parents overweight	558	387	69.4
	One or both parents immigrants/foreign nationals^e^	565	77	13.6
	Household size in m^2^ per person (mean) (SD)	562	38.0 (16.6)	
	TV in child's bedroom	598	3	0.5

a *Excluding mothers under 18 years of age for KUNO Kids birth cohort*.

b *Based on BMI > 25 kg/m^2^, mothers at one year after delivery, fathers at four weeks after delivery*.

c *Based on the MacArthur Scale of Subjective Social Status; numbers range from 1–10*.

d *Based on extent of professional qualification (“None”: no finished education, not in professional education,” Low”: student or still in professional education, “Medium”: completed education, under university level, “High”: university education)*.

e *Born in a foreign country or with a foreign passport*.

### Pattern and Amount of Exposure to Different Digital Media During First Year of Life

In general, 45% of the 12-month-old children did not meet the AAP guidelines of absolutely no screen time.

**Figure 1:**
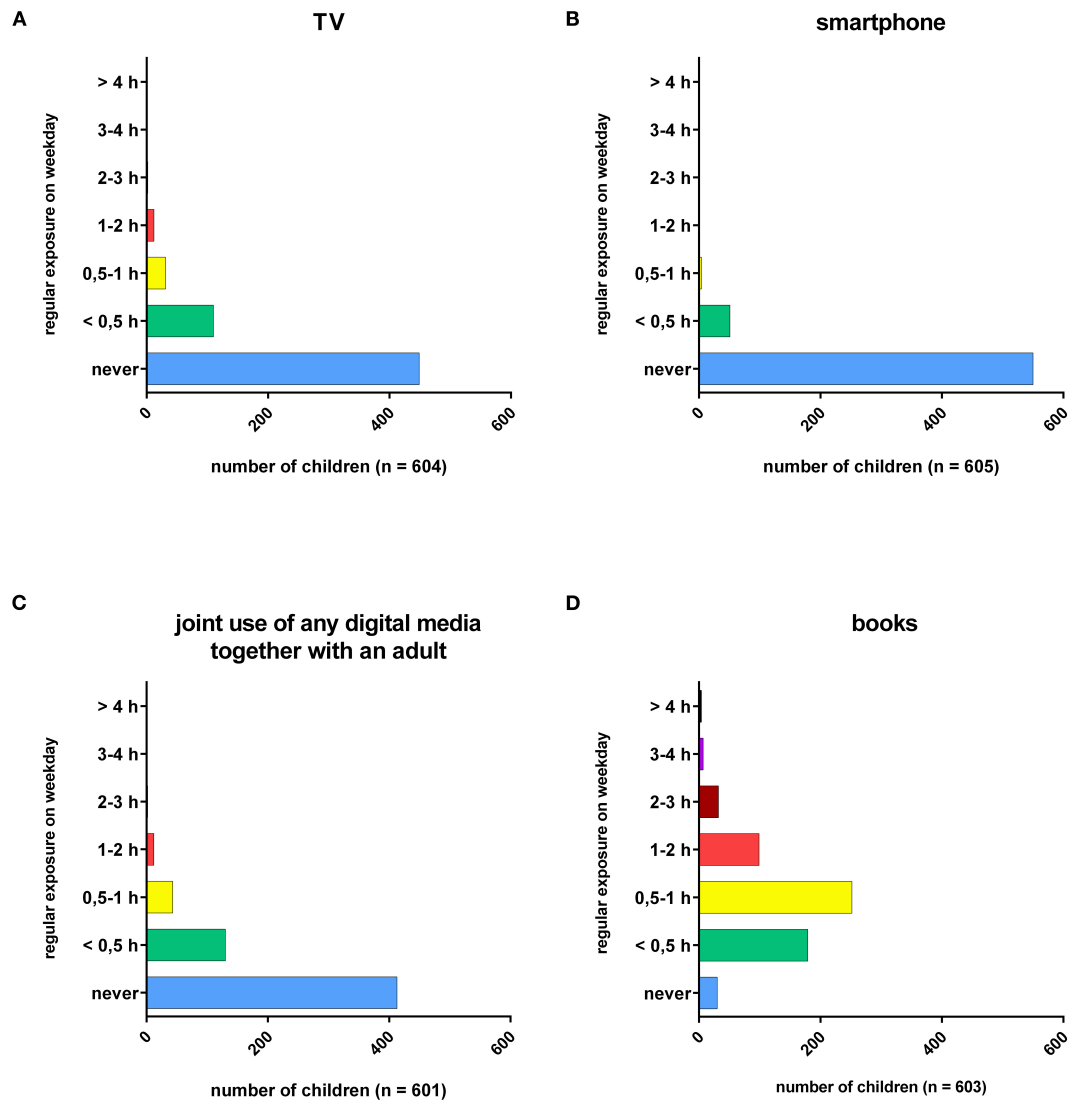
Regular exposure time (in hours) to different types of media on weekdays.

On an average weekday, most infants did not watch TV or use smartphones, tablets, Blu-ray/DVD/Video, game consoles or PCs (ranging from 74–99.6%). However, there was a subgroup of children who were regularly exposed to electronic media, most commonly TV (Figure 1A) or smartphone (Figure 1B), for up to 0.5 hper day (TV: 20 % (110), *n* = 604; smartphone: 9 % (51), *n* = 605). Joint media use was a little higher (Figure 1C). 31% of parents reported using digital media together with their child, usually for up to 0.5 h on a typical weekday (130, *n* = 601). Reading books together with children was even more popular (Figure 1D). Most parents and infants read up to 1 h per day together (71.5 %, 431, *n* = 603). Only 5% did not spend any time at all using books.

**Figure 2:**
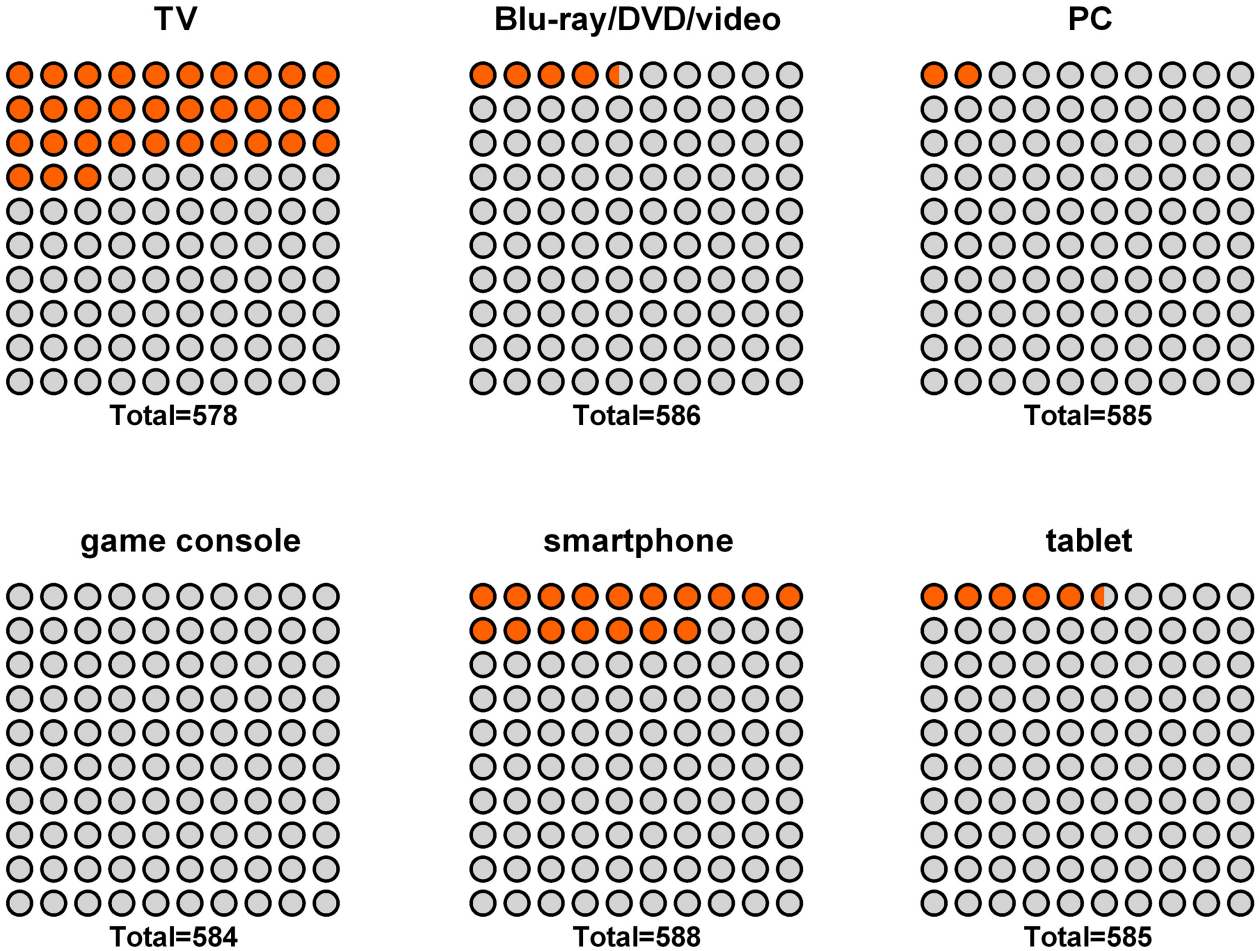
Percentage of infants exposed to different types of media during the first year of life.

When examining the particular digital media devices, it became clear that 33.0% of children had been exposed to TV, 16.9% to smartphones, 5.3% to tablets, 4.4% to Blu-ray/DVD/video, 2.2% to PC and 0.2% to game consoles at least once in the first year of life (Figure 2). The age of earliest contact was commonly around 7–9 months (TV, smartphone, tablet, Blu-ray/DVD/video, PC) (Table 2). The few children who were exposed to game consoles were about 12 months old.

**Table 2:** Age in months at first exposure to different digital media.

**Device**	**Mean**	**Median**	**SD**	**Min**	**Max^a^**	***n***
TV	8.3	9.0	2.8	1	13	192
Blu-ray/DVD/Video	7.7	8.0	3.0	1	13	27
Tablet	8.3	9.0	3.1	2	12	31
Smartphone	8.6	10.0	2.6	1	13	100
Game console	12.0	12.0	-	12	12	1
PC	8.2	8.0	2.1	6	12	13

a *Maximum of 13 months of age is possible as questionnaires were included that were filled out up to 2 months after the child's first birthday*.

### Family TV Time (Passive Child Media Exposure)

**Figure 3:**
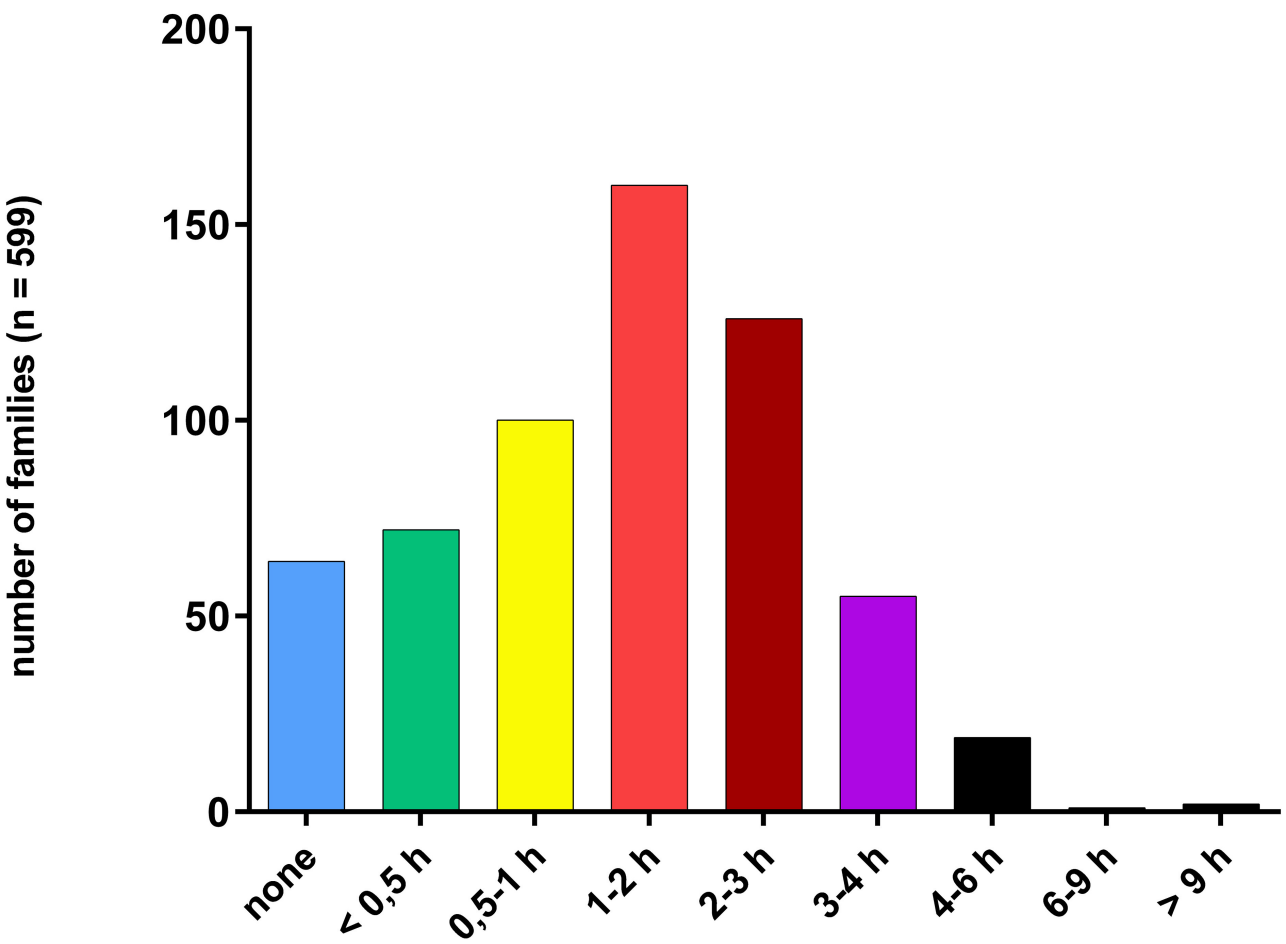
Family TV time (in hours) on average weekday.

The results of household TV use showed a Gaussian distribution curve, with a peak at 1–2 h of TV screen time on an average weekday (27%, 160, *n* = 599) (Figure 3). 21% of parents had a TV running for 2–3 h and 17% between 0.5–1 h. Only 11% stated that they did not watch TV on a regular daily basis.

### Factors Associated With Infant's Exposure to Digital Media

In both the univariable (Table 3) and multivariable analysis (Table 4), household space per person (multivariable analysis: *p* = 0.007, OR = 0.567, 95% CI = 0.375–0.857) and excessive family TV time (multivariable analysis: *p* = 0.027, OR = 1.631, 95% CI = 1.059–2.512) showed a significant association to the child's media exposure. Family television time of 2–3 h or more per day raised the odds by 63% (multivariable data) of the child having screen time. More living space per person than the median indicated a 43% less chance of child screen time. While the univariable analysis revealed the mother's subjective social status as a predicting factor (*p* = 0.001, OR = 0.795, 95% CI = 0.696–0.909), in the multivariable analysis this association closely missed the level of significance (*p* = 0.058, OR = 0.853, 95% CI = 0.723–1.005) and was no longer independently associated with the infant's screen time. The multivariable analysis uncovered a significant relationship between the presence of a sibling and child screen time (*p* = 0.019, OR = 1.715, 95% CI = 1.093–2.692). Being second-born increased the chance of screen time by 72%.

**Table 3:** Univariable logistic regression analysis of possible predictors and infant screen time.

		**Odds ratio**	**CI 95%**	**Significance (*p* value)**
Infants	Sex female	0.784	0.560–1.098	0.156
	1 sibling^a^	1.443	0.998–2.085	0.051
	2 or more siblings^a^	1.209	0.621–2.353	0.577
Mothers	Age at delivery (years)	0.968	0.928–1.010	0.138
	Subjective social status	0.795	0.696–0.909	**0.001**
	Single parent	0.307	0.034–2.765	0.292
	Professional qualification low^b^	1.333	0.113–15.704	0.819
	Professional qualification medium^b^	0.243	0.027–2.199	0.208
	Professional qualification high^b^	0.161	0.018–1.464	0.105
	Employed during the child's first year of life	0.969	0.664–1.414	0.871
Fathers	Subjective social status	0.940	0.834–1.058	0.305
	Professional qualification low^c^	0.333	0.023–4.736	0.417
	Professional qualification medium^c^	0.331	0.034–3.223	0.341
	Professional qualification high^c^	0.208	0.021–2.032	0.177
	Employed during the child's first year of life	1.418	0.470–4.278	0.535
Parents	Overweight (one or both)	1.318	0.909–1.911	0.146
	One parent immigrant/foreign national^d^	1.223	0.699–2.140	0.481
	Both parents immigrants/foreign nationals^d^	1.268	0.518–3.105	0.603
Home	Living space^e^	0.610	0.433–0.860	**0.005**
	Excessive family TV time^e^	1.513	1.053–2.173	**0.025**

a *Reference category: first-born*.

b *Reference category: no maternal professional qualification*.

c *Reference category: no paternal professional qualification*.

d *Reference category: both parents German nationals*.

e *As defined in materials and methods. Bold values indicates significant association (p <0.05) in the uni/multivariable analysis*.

**Table 4:** Multivariable logistic regression analysis of possible predictors and infant screen time^*^ (*N* = 441, Nagelkerkes-R^2^ = 0.108).

		**Odds ratio**	**CI 95%**	**Significance (*p* value)**
Infants	Sex female	0.721	0.485–1.072	0.106
	1 sibling^a^	1.715	1.093–2.692	**0.019**
	2 or more siblings^a^	1.333	0.557–3.193	0.519
Mothers	Age at delivery (years)	0.974	0.920–1.032	0.373
	Subjective social status	0.853	0.723–1.005	0.058
	Professional qualification low^b^	0.151	0.007–3.480	0.238
	Professional qualification medium^b^	0.474	0.045–5.016	0.535
	Professional qualification high^b^	0.387	0.036–4.176	0.434
Fathers	Professional qualification low^c^	0.169	0.010–2.890	0.220
	Professional qualification medium^c^	0.212	0.019–2.406	0.211
	Professional qualification high^c^	0.161	0.014–1.811	0.139
Parents	Overweight (one or both)	1.162	0.753–1.793	0.496
Home	Living space^d^	0.567	0.375–0.857	**0.007**
	Excessive family TV time^d^	1.631	1.059–2.512	**0.027**

* *Including the independent variables with p <0.2 in the univariable analysis*.

a *Reference category: first-born*.

b *Reference category: no maternal professional qualification*.

c *Reference category: no paternal professional qualification*.

d *As defined in materials and methods. Bold values indicates significant association (p <0.05) in the uni/multivariable analysis*.

The other potential risk factors examined did not show statistically significant associations with child screen time. Sensitivity analyses with screen time <0.5 h vs. >0.5 h were conducted for the univariable logistic regression analysis and revealed similar results.

## Discussion

The purpose of this study was to report baseline data of young children's digital media use and explore potential risk factors for exposure. Contrary to AAP and national guidelines' recommendations, about half the children have already been exposed to digital media during the first year of their life. While most infants did not have regular screen time, there was a subset of 10% of the study population who had a regular exposure for up to half an h on an average weekday. The usual first contact to digital media occurred at the age of 7–9 months. In addition, the presence of a sibling, less personal living space, and increased family TV time were found to be significant predictors of child screen time.

For the examined age group in our present study, to best of our knowledge, hitherto no data exist. However, previous studies on media consumption in older children reported a higher exposure. For example, the 2017 Common Sense Media survey showed that among American children under 2 years of age, 71% had been exposed at least once to TV, 45% to DVD/video, and 46% to mobile devices (20). In Singapore, 24% of infants up to 6-months-old had TV screen time, rising to 61% of 7–24-month-old infants. When viewing the screen time of TV, computer, and mobile devices together, one third of 6-month-old infants had screen time opposed to two thirds of the older infants (21). Together with our data, this might illustrate a trend of increased child screen time with age, as already corroborated by Duch et al. in their review (22).

Not only are children using digital media devices at an earlier age (22), but they are also exposed to a multitude of new interactive media devices (3). Nearly twenty years ago, Vandewater et al. published results showing 0–3-year-olds having an average screen time of nearly 2 hours per day, including TV, videos/DVDs, computers and video games (23). In 2007, Zimmerman et al. only considered TV and DVD/video viewing time at 2 years of age for American children (24). When looking at the German population, the ULM Spatz birth cohort reported 58% of three-year-old children watching TV/DVDs as well as using smartphones up to 1h/day and 14% for more than 1h (screen time data gathered from 2014–2016) (25). Yet, these studies do not name or differentiate between the different types of electronic media available today.

In turn, the Common Sense Media survey divided digital media use by content. The report stated that 0–2-year-olds spent on average, 40 min of TV/DVD/video time (including videos watched on mobile devices), 21 min reading/being read to and 0 min playing games on mobile devices. Also, although average child digital media time remained about the same between 2011 and 2017, a shift toward new mobile devices became apparent (20). Our data, showing initiation of digital media use primarily with TV and smartphone, could be an indicator of a similar trend beginning among infants in Germany.

Adding to evidence, we show that family TV screen time, living space per person, and being second-born are significant predictors of infant screen time. In 2015, Kabali et al. noted that 97 % of children (0.5–4 years old) from low-income minority families in the US had ever used a mobile device and most watched TV daily regardless of age (1). This is consistent with findings of the Common Sense Media report, showing that children from lower income households had substantially more screen time per day (3:29 h vs. 1:50 h, age group 0–8 years). Lower education and ethnic minority (African American > Hispanic > White) were also associated with significantly more child media time. Similarly, a systematic review by Duch et al. showed positive associations between screen time and child's age, BMI, and family belonging to a minority population in children between 0 and 36 months of age (22). Tandon et al. found that children of families with low socioeconomic status had more electronic media devices in the bedroom and more screen time than families with higher socioeconomic status (26). In our multivariate analysis of influencing factors for mean screen time of the infant, we saw a tendency for the maternal subjective social status (MacArthur Scale) to be a protective factor (OR 0.853, *p* = 0.058). Among other factors with a clear association to socioeconomic status, we found the family's living space to have a strong association with the infant's screen time (OR 0.567, *p* = 0.007).

In our study, a further influencing factor with a significant association to infant's screen time was the family TV time (OR 1.631, *p* = 0.027). Interestingly, Jago et al. showed that maternal TV viewing was a stronger predictor of child TV viewing than paternal TV viewing for all age (<7 and >7 years) and gender subgroups (16). It should be noted that these associations are made from cross-sectional data, therefore the direction of the relationship between infant and family media ecology as well as sociodemographic characteristics remains to be investigated.

For young infants, the displacement of activities such as sleep and play by screen media may be particularly harmful toward their behavioral, physical, and cognitive development, not to mention their communication abilities (12). A study by Twenge et al. examined screen time and sleep duration in children from 0–17 years. Their findings showed that both portable and non-portable electronic devices influenced sleep duration for children under age 10 (once over the age of 10, only portable devices had an effect on sleep) (27). This supports the assumption that screen time displaces valuable childhood activities. As numerous studies show, reading or being read to by parents can be supportive in language development (both maternal and foreign language abilities) (28), socio-emotional development (29) and even obesity (30) in young children. Promoting adult-child reading time may be an important protective measure against excessive screen time and stimulate positive child development.

It is important to consider the limitations of our study. First, the study design as a birth cohort with above mentioned exclusion criteria caused a selection bias. Participants are predominantly of German nationality and must be proficient German speakers. In addition, average subjective social status as a proxy for socioeconomic status is relatively high. Second, social desirability bias might play a role when parents state the family's and child's media exposure. Radesky et al. showed that 1/3 of parents underestimated and 1/3 of parents overestimated their child's amount of media use, when comparing online questionnaire to passively measured screen time via an app (31), an observation which indicates that over- and underestimation might be in a balance. Future research will provide more reliable data by directly measuring the time spent with electronic media devices in the homes. Third, in our case of 1-year-old children, child media exposure might simply be passive or background media, if for example infants are with parents while they themselves are using digital media. Still, we did not determine content or motivation of child media use at this young age and can only speculate. Lastly, as a cross-sectional study, the causal direction of the relationship between associated factors and screen time cannot be determined.

In conclusion, our study provides support that excessive family TV time is a major predictor of infant screen time. Moreover, we found smaller living spaces and having one sibling to be significant risk factors. At the age of 12 months, a proportion of 10% of the study population was already regularly exposed to digital media up to half an hour per day. Prospective studies should investigate the effect of passive media exposure, such as background TV. In addition, because child development changes rapidly in the first years, it is crucial to examine younger children and smaller age groups. We suggest that pediatric recommendations should be re-evaluated in the light of the actual exposure to digital media already in infancy and parents should be proactively counseled regarding possible effects on child development.

## The Kuno Kids Study Group

The members of the KUNO Kids study group are: Andreas Ambrosch (Institute of Laboratory Medicine, Microbiology and Hygiene, Barmherzige Brüder Hospital, Regensburg, Germany); Petra Arndt (ZNL Transfercenter of Neuroscience and Learning, University of Ulm, Ulm, Germany); Andrea Baessler (Department of Internal Medicine II, Regensburg University Medical Center, Regensburg, Germany); Mark Berneburg (Department of Dermatology, University Medical Centre Regensburg, Regensburg, Germany); Stephan Böse-O'Reilly (University Children's Hospital Regensburg (KUNO), Hospital St. Hedwig of the Order of St. John, Regensburg, Germany); Romuald Brunner (Clinic of Child and Adolescent Psychiatry, Psychosomatics and Psychotherapy, Bezirksklinikum Regensburg (medbo), Regensburg, Germany); Wolfgang Buchalla (Department of Conservative Dentistry and Periodontology, University Hospital Regensburg, University of Regensburg, Regensburg, Germany); Sara Fill Malfertheiner, Sebastian Häusler (Clinic of Obstetrics and Gynecology St. Hedwig, University of Regensburg, Regensburg, Germany); André Franke (Institute of Clinical Molecular Biology, Christian-Albrechts-University of Kiel, Kiel, Germany); Iris Heid (Department of Genetic Epidemiology, University of Regensburg, Regensburg, Germany); Caroline Herr (Bavarian Health and Food Safety Authority (LGL), Munich, Germany); Wolfgang Högler (Department of Pediatrics and Adolescent Medicine, Johannes Kepler University Linz, Linz, Austria); Sebastian Kerzel (Division of Pediatric Pneumology and Allergy, University Children's Hospital Regensburg (KUNO), Hospital St. Hedwig of the Order of St. John, Regensburg, Germany); Michael Koller (Center for Clinical Studies, University Hospital Regensburg, Regensburg, Germany); Michael Leitzmann (Department of Epidemiology and Preventive Medicine, University of Regensburg, Regensburg, Germany); David Rothfuß (City of Regensburg, Coordinating Center for Early Interventions, Regensburg, Germany); Wolfgang Rösch (Department of Pediatric Urology, University Medical Center, Regensburg, Germany); Bianca Schaub (Pediatric Allergology, Department of Pediatrics, Dr. von Hauner Children's Hospital, University Hospital, LMU Munich, Munich, Germany); Bernhard H.F. Weber (Institute of Human Genetics, University of Regensburg, Regensburg, Germany); Stephan Weidinger (Department of Dermatology, Venereology and Allergy, University Hospital Schleswig-Holstein, Kiel, Germany); Sven Wellmann (Division of Neonatology, University Children's Hospital Regensburg (KUNO), Hospital St. Hedwig of the Order of St. John, Regensburg, Germany).

## Data Availability Statement

The raw data supporting the conclusions of this article will be made available by the authors, without undue reservation.

## Ethics Statement

The studies involving human participants were reviewed and approved by Ethics Committee of the University Regensburg. Written informed consent to participate in this study was provided by the participants' legal guardian/next of kin.

## Author Contributions

KD wrote the manuscript and designed the tables. DW was responsible for the recruitment of study participants, data collection, and validation. SB performed the statistical analyses. BS-G, CA, MM, and MK contributed to the design of the study, the interpretation of the results, and the authoring of the manuscript. SK developed the study question and design, supervised the data analysis, participated in writing the manuscript, and created the figures. All authors contributed to the article and approved the submitted version.

## Conflict of Interest

The authors declare that the research was conducted in the absence of any commercial or financial relationships that could be construed as a potential conflict of interest.

## Publisher's Note

All claims expressed in this article are solely those of the authors and do not necessarily represent those of their affiliated organizations, or those of the publisher, the editors and the reviewers. Any product that may be evaluated in this article, or claim that may be made by its manufacturer, is not guaranteed or endorsed by the publisher.
